# The biomechanical implications of neck position in cervical contusion animal models of SCI

**DOI:** 10.3389/fneur.2023.1152472

**Published:** 2023-06-06

**Authors:** Numaira Obaid, Kazuhito Morioka, Eleni Sinopoulou, Yvette S. Nout-Lomas, Ernesto Salegio, Jacqueline C. Bresnahan, Michael S. Beattie, Carolyn J. Sparrey

**Affiliations:** ^1^Mechatronic Systems Engineering, Simon Fraser University, Surrey, BC, Canada; ^2^International Collaboration on Repair Discoveries (ICORD), Vancouver, BC, Canada; ^3^Department of Orthopaedic Surgery, University of California, San Francisco, San Francisco, CA, United States; ^4^Brain and Spinal Injury Center, Department of Neurological Surgery, University of California, San Francisco, San Francisco, CA, United States; ^5^Center for Neural Repair, University of California, San Diego, San Diego, CA, United States; ^6^Department of Clinical Sciences, Colorado State University, Fort Collins, CO, United States; ^7^University of California, Davis, Davis, CA, United States

**Keywords:** spinal cord injury, surgical positioning, contusion models, animal models, contusion injury

## Abstract

Large animal contusion models of spinal cord injury are an essential precursor to developing and evaluating treatment options for human spinal cord injury. Reducing variability in these experiments has been a recent focus as it increases the sensitivity with which treatment effects can be detected while simultaneously decreasing the number of animals required in a study. Here, we conducted a detailed review to explore if head and neck positioning in a cervical contusion model of spinal cord injury could be a factor impacting the biomechanics of a spinal cord injury, and thus, the resulting outcomes. By reviewing existing literature, we found evidence that animal head/neck positioning affects the exposed level of the spinal cord, morphology of the spinal cord, tissue mechanics and as a result the biomechanics of a cervical spinal cord injury. We posited that neck position could be a hidden factor contributing to variability. Our results indicate that neck positioning is an important factor in studying biomechanics, and that reporting these values can improve inter-study consistency and comparability and that further work needs to be done to standardize positioning for cervical spinal cord contusion injury models.

## 1. Introduction

Spinal cord injuries (SCIs) impact 250,000 to 500,000 people each year (1), often with catastrophic outcomes, particularly when the injury occurs at the cervical spinal level. The prevalence of SCIs, coupled with the resulting functional loss, prompts an urgent need to develop treatments. Evaluating and developing treatments for human SCI relies on experimentally reproducing the patterns of tissue damage and neurological deficits that are observed in human SCIs in animals. Contusion models of SCI in animals are one of the most clinically relevant models since they best mimic a human spinal cord injury (2).

Since a traumatic SCI occurs from a mechanical event, the tissue damage patterns and functional deficits after an SCI stem from the biomechanics of the event. This makes it important to understand how an external impact to the spine distributes stress and strain across the various constituent tissues of the cord, resulting in their damage. Understanding the interrelationships between impact biomechanics, tissue damage, and functional deficits provides a foundation to develop more consistent animal injuries to evaluate SCI treatment efficacy.

The tissue-level biomechanics and tissue damage patterns produced in animal contusion models are sensitive to various parameters (3–5). In the past, sources of experimental variability in contusion injury models, including impact velocity (6), displacement (7), force (8), energy (3), and spinal level have been identified, and as a result, these impact parameters are measured and reported in many experimental studies. Several other parameters, such as impactor size and subject characteristics (weight, age), have even been standardized to improve consistency and comparability between different studies (9).

Experimental variability continues to be observed in large animal contusion models of spinal cord injury (3, 10, 11). Further improving experimental consistency requires examination of the entire experimental process to ensure that the impact and resulting tissue damage is as consistent as possible between different subjects in the same study. Additionally, as more evidence is accumulated and more experimental parameters are standardized, there is less experimental dispersion, which improves reproducibility and facilitates comparison between different studies.

One area that has not been examined in greater detail is how the head and neck of the animal should be positioned, and how positioning could impact the outcomes and variability in animal SCI models. While head and neck positioning are well-recognized for their significant effects on human spinal cord morphology and spinal cord injury biomechanics (12, 13), there is limited exploration of its effects in animal contusion models. In fact, positioning of the head and neck of animals is often under-reported and is far from being standardized in animal models of SCI.

In this study, we will review the effect of neck position on the spinal cord over the cervical range of motion in humans and use that to discuss how neck position could impact outcomes and variability in cervical spinal cord contusion injuries in animal studies. The affect of neck position on the cervical spinal cord in animals has been demonstrated in prior studies (14, 15). However, extensive studies over the cervical spine range of motion are quite limited in animals, therefore, our paper will primarily summarize the neck flexion/extension and spinal cord relationships previously explored in human subjects, and we discuss the application and implications in large animal models of SCI.

## 2. Biomechanics of contusion models of SCI

Experimental spinal cord contusion injuries are generated from the dorsal side of the spinal cord following surgical exposure. Briefly, subjects are administered anesthesia and are then placed in a prone position, often in a stereotaxic frame. A surgical incision is made, and the spinal muscles are retracted. A laminectomy or partial laminectomy exposes the spinal cord, and a contusion injury is delivered to the dura over the dorsal surface of the spinal cord. Following the impact, the incision is closed in anatomical layers, and the subject recovers (16, 17).

There are different techniques used to deliver a contusion impact and cause damage to the cord tissues, each having different biomechanical considerations. In a weight drop impactor, a known mass is dropped onto the spinal cord from a pre-determined height, resulting in potential energy transfer from the mass to the cord (2, 8). In a force-controlled impactor (such as the Infinite Horizons impactor) (8, 18), the cord is displaced by an impactor until the measured force reaches a target pre-set force value, and in a displacement-controlled impactor (such as the Ohio State University impactor or Bose impactor) (4, 19), the maximum displacement of the impactor is controlled, and the resulting force is record for each subject.

During a contusion impact, the instantaneous compression of the cord results in damage to the constituent tissues of the cord. The external impact distributes across the constituent tissues based on their mechanical properties and geometry (10, 11, 20–22). Since the cord is inherently heterogeneous in both the mechanical properties and geometries of the different tissues, an external load causes different regions of the cord to experience different levels of stress and strain (11, 23, 24). When the stress/strain in a specific region exceeds a threshold value, the tissue becomes damaged (11). Patterns of stress/strain distribution in the spinal cord is an indicator of tissue damage patterns, which are related to the functional deficits observed after a spinal cord injury occurs (11, 23–25).

Tissue-level stress/strain patterns within the cord stem from various biomechanical factors including external factors such as the surgical procedure, and impact parameters (such as speed and displacement) (6–8). These factors have been well-investigated and are often the kept consistent between different studies. Recent studies have also highlighted the importance of internal, or subject-specific, factors on tissue-level biomechanics. These factors include the mechanical properties of the constituent tissues (10, 21, 26–29), and the transverse morphology of the spinal cord and canal of the individual subject (such as the cord mediolateral and dorsoventral diameter (3, 22), and the thickness of CSF surrounding the spinal cord (30)).

## 3. Effects of neck positioning in animal models

To the best of our knowledge, there are no studies that explore how the neck/head of the animal should be positioned while a contusion injury is delivered, most studies aim for a neutral position with limited flexion or extension at the level of injury. However, positioning away from the injury site may receive less attention. Based on prior studies conducted in humans, the neck position plays an important role on the biomechanics of a spinal cord injury (31, 32). Extrapolating from human studies, three potential effects that neck positioning could have on animal models are: (1) it could affect the exposed spinal level, (2) it could change the transverse morphology of the spinal cord, and (3) it could alter the mechanical properties of the constituent tissues.

### 3.1. Effect on exposed spinal level

The contused spinal cord level impacts the functional deficits that are experimentally observed in the animal since functional impairments occur at and below the injury site (33). Each vertebral level of the cervical spine is responsible for different functional abilities, and the level at which the tissue damage occurs dictates the specific functional deficits that occur after SCI. The relationship between spinal level and functional deficits is important because studies have shown that the spinal cord moves in the caudal/rostral direction depending on neck position. Therefore, not ensuring consistent positioning can lead to differences in the precise neurological level of injury, causing variability in observed functional outcomes. Ensuring that the correct spinal level is exposed is particularly critical at the cervical enlargement of the cord, where the motor control and innervation for the upper limbs are processed in the spinal cord gray matter (34).

The rostral/caudal movement of the spinal cord with neck positioning is not well-examined for animal subjects; however, earlier work indicates that relative to the spinal canal, flexion of the neck in humans caused the dura and spinal cord to move 1.5 mm rostrally at the C5 vertebral level, and 2.7 mm rostrally at the C6 vertebral level (35). Later studies used MRI to measure *in vivo* displacement of the cervical spinal cord at different neck positions, and showed that during flexion and extension, different cervical levels experience different amounts of rostral/caudal movement (Table 1A) (36). Yuan et al. (1998) also showed that within the cervical region, different segments of the cord moved in different directions under neck flexion, i.e. the lower cervical cord moved cranially, while the upper cervical cord moved caudally (37). This study also reported differences between the displacement of the dorsal and ventral surfaces of the cervical spinal cord at different levels under neck flexion (Table 1B), where the dorsal surface of the cord experienced higher displacement.

Table 1The effect of flexion on spinal level.

**Table:** 

**(A)**	**C3**	**C4**	**C5**	**C6**	**C7**
**Neck flexion**	−0.38	−0.74	−0.94	1.80	1.06
**Neck extension**	−5.56	−0.70	−0.79	−0.98	−2.18

**Table:** 

**(B)**	**C2**	**C3**	**C4**	**C5**	**C6**	**C7**
**Ventral**	−1.5	−1.1	−0.5	−0.4	0.9	2.1
**Dorsal**	−2.7	−2.6	−1.5	−0.9	1.1	2.7

^*^Measurements are reported in millimeters. Negative measurements represent a caudal movement, positive values represent a rostral movement. (A) Neck flexion or extension causes the spinal cord to move in the caudal or rostral direction relative to the spinal canal, based on the spinal level, reproduced from Margulies et al. (36). (B) Neck flexion causes different displacements in the dorsal vs. ventral surfaces of the spinal cord, reproduced from Yuan et al. (37).

The same principles investigated in these studies can have implications on animal contusion models as well. A study by Smith, which explored the effects of neck position in the cervical spine of the *Macaca mulatta* (rhesus monkeys), demonstrated the importance of consistent neck position in non-human primate contusion injuries (14). This study reported that under flexion, different segments of the spinal cord shift in the caudal/rostral directions with C1–C4 displacing 1.6, 1.3, 0.9, and 0.2 mm caudally, respectively. The study observed that flexion of both the head and the trunk together can alter the relationship between the vertebral canal and the spinal cord such that the segment of the spinal cord corresponding to a vertebral level is located higher than expected despite the dentate ligaments limiting its range. This means that due to the position-induced rostral movement of the cord, if neck position is not controlled in a contusion experiment, the specific spinal level receiving an injury could be different between individual subjects. Since the specific contused spinal level affects functional outcomes, variability in the level of a lesion could alter the functional outcomes observed in a study. Thus, position-dependent spinal cord displacement is a considerable factor for the reproducibility in cervical contusion experiment.

### 3.2. Effect on spinal cord morphology

The functional deficits resulting from SCI are dependent on the location and extent of tissue damage after an impact. These patterns of tissue damage are correlated with the strain distribution produced in the constituent tissues during the impact, i.e., the tissue-level damage is correlated with how an external impact creates local deformation and stress in the individual tissues (10, 20, 24, 25). Experimental studies show that despite applying a consistent impact protocol to all subjects in an experimental study, there is inter-subject variability in the tissue damage observed via histology, indicating that there may also be variability in the tissue-level biomechanics between subjects.

The morphology of the spinal cord and canal have been identified as important factors influencing tissue-level strain distributions and patterns of tissue damage after SCI. Kim et al. (3) conducted a statistical analysis between spinal cord morphometry, biomechanics, and functional outcomes in a porcine model of thoracic contusion SCI. The study showed a strong correlation between impact biomechanics and cord morphology and identified that a small dorsoventral diameter of the thoracic cord correlated with poorer functional outcomes after SCI (3). This study also highlighted the importance of the ventral cerebrospinal fluid (CSF) space in tissue sparing and behavioral outcomes (3). Nishida et al. (22) used finite-element modeling to show that morphological differences between a human spinal cord at the C2-C7 level altered the tissue-level stresses under moderate compression in a simulated unilateral SCI model (22). Fournely et al. (38) used finite-element analysis to highlight the importance of the spinal cord diameter on the strain levels in the spinal cord during a contusion impact (38). Several studies have also shown that the amount of CSF affects the biomechanics of spinal cord injuries (30, 39). Persson et al. (30) investigated the importance of cerebrospinal fluid thickness on the biomechanics of traumatic spinal cord injury through a computational model and concluded that decreased CSF thickness caused higher stresses in the cord during deformation (30).

**Figure 1:**
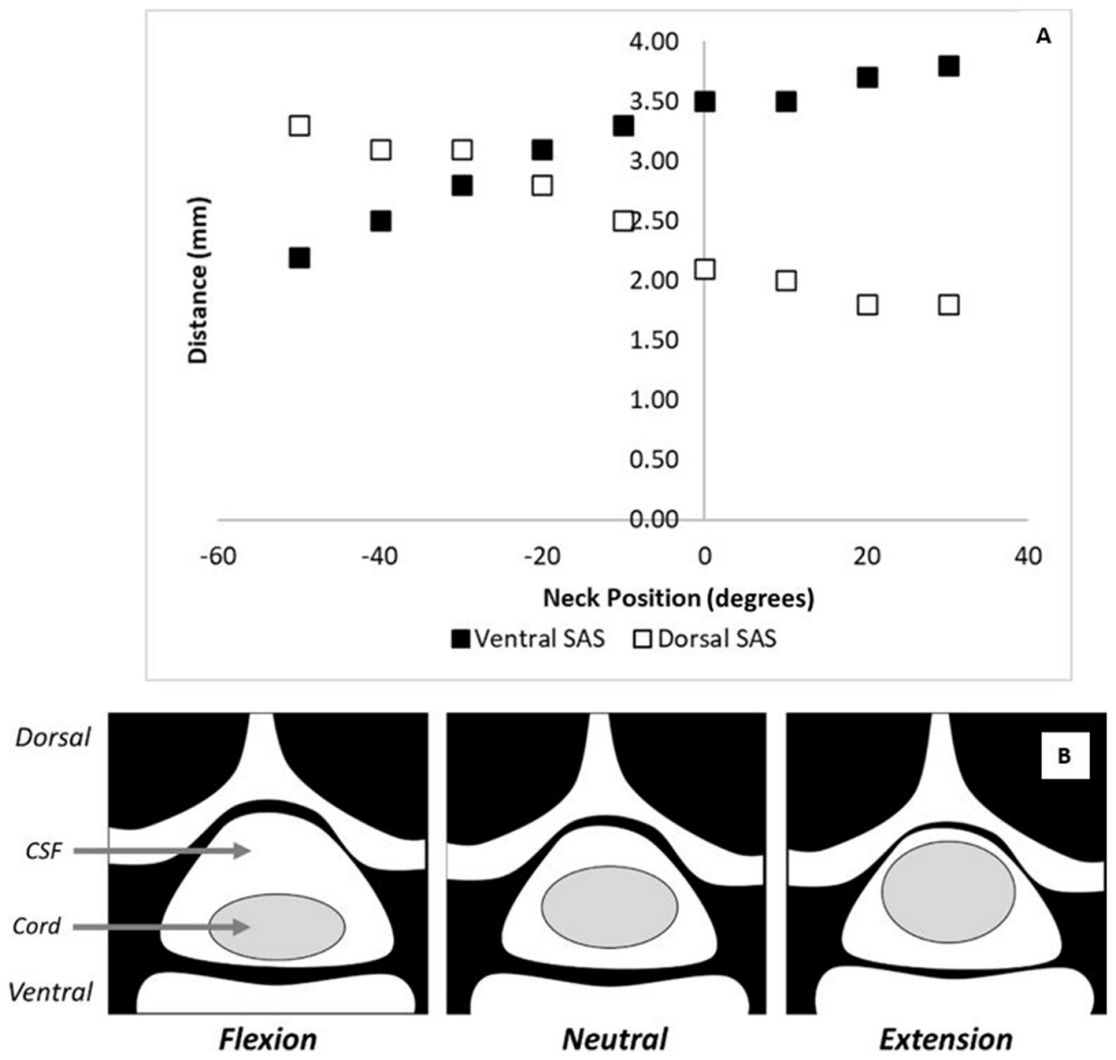
The effect of flexion on spinal cord morphology. **(A)** The ventral and dorsal subarachnoid space changes with neck position as reported by Muhle et al. (40) at the C5 level. In this figure, negative values on the x-axis represent degrees of flexion and positive values represent degrees of extension. **(B)** A schematic showing the cord/canal morphology under various neck positions.

The role of morphology on tissue-level biomechanics is significant when investigating why the position of the head and neck of an animal subject might be important during a contusion injury. Morphological changes in the cervical spine at different neck positions has been previously quantified (Figure 1A) (40). Compared to a neutral posture, extension of the neck increased the dorsoventral diameter of the spinal cord while flexion decreased the cord diameter (Figure 1B). In addition, under supine flexion, the spinal cord shifts ventrally while under extension, the cord shifts dorsally (40, 41). These observations were confirmed in other studies, where it was also reported that the dorsoventral diameter of the spinal cord flattens under flexion and expands under extension (41). These studies indicate that during flexion, the spinal cord occupies less space in the canal than under neutral or extended positions due to cord compression. Under flexion, the cord is also flatter (oblique) than in neutral posture. Under neck flexion, the pressure in the cerebrospinal fluid increases, which can also alter the injury biomechanics (42).

These morphological changes in the spinal cord and spinal canal can have significant implications for the biomechanics of a contusion injury. Inter-subject variability in neck position will produce variability in the dorsoventral diameter of the cord during the impact. The dorsoventral diameter of the cord impacts both tissue biomechanics and tissue damage; therefore, this inter-subject variability could lead to variability in tissue damage patterns and observed functional outcomes as well. Additionally, the ventral shift of the spinal cord during flexion would decrease the amount of CSF between the cord and the spinal canal. Since the CSF normally protects the cord from impact against the spinal canal (43), a decrease in its thickness could alter the tissue-level biomechanics of a contusion injury.

Flexion of the neck also increases the pressure exerted by the spinal cord onto the ventral wall of the canal, resulting in contact forces between the canal and the meninges (35, 44). A previous study measured the force required to lift the spinal cord from the canal in the cervical region in human cadavers, and observed that under flexion, there was a ten-fold increase in the force required to move the C5–C6 segment of the spinal cord in the dorsoventral direction (35). This indicates a significant change in the pretension of the spinal cord and meninges. This means that if the neck is placed under flexion during a contusion injury, it could increase the possibility of the cord slipping laterally during unilateral contusion experiments (18, 19). If flexion is required during the surgical procedure like laminectomy, it is recommended that the subject be repositioned in a neutral posture prior to impact but further investigation is required.

### 3.3. Effect on mechanical properties of tissues

The impact applied during a contusion injury distributes to the constituent tissues of the spinal cord including gray and white matter, pia mater, the CSF, and dura mater. The degree of stress and strain in each spinal tissue is dependent on its individual mechanical properties (20, 28). Most computational models assume the meninges (dura, arachnoid, and pia mater) to be linear elastic materials, which means, that the degree of deformation in these tissues is linearly proportional to the applied load at low strains. More recently, the material properties and relaxation have been shown to be dependent on strain rate (10, 20, 45). In addition, the dura mater exhibits nonlinear material properties (46). Experiments on white matter and gray matter tissues showed hyper viscoelastic behavior (20, 21, 26, 28). Unlike linear elastic materials, the stiffness, or modulus, of the gray and white matters change based on the amount of strain applied to these materials (27, 47, 48). Altering the relative stiffness of the constituent tissues changes how an applied contusion impact distributes to the constituent tissues and induces different patterns of tissue damage during a spinal cord injury (20, 28).

Neck position has important implications for the mechanical properties of the constituent tissues due to tissue pretension. Neck flexion increases the length of the vertebral canal, and consequently, stretches the dura mater (26, 49, 50). It is believed that because the spinal cord is connected to the dura mater via the dentate ligaments, the flexion-induced dural stretching causes the spinal cord to stretch as well (49, 50). The spinal cord extends by ~2–10% under neck flexion (32, 35, 37, 51). Similarly, the spinal cord decreases in length under extension. Breig et al. (52) estimated that under neck extension, the spinal cord decreases in length by approximately 8-10 mm compared to a neutral posture (52) and Kuwazawa et al. (51) reported a decrease in length of 3–6% under neck extension (51).

Smith (14) explored the effect of neck position on spinal cord elongation in Macaca mulatta, and reported that under flexion, the cord elongated proportional to the flexion of the corresponding joint (14). When the neck and head were flexed, the spinal cord elongated by 16% at the atlanto-occipital joint (C0/C1 level), 9% at the C2/C3 level, and 25% at the C6/C7 level. Furthermore, Smith (14) showed that even if the neck is in a neutral posture, the entire length of the spinal cord stretches by hip flexion, knee extension, and foot dorsiflexion. Similarly, stretching of the hand causes the brachial nerves to pull on the lower cervical spinal cord. Since the entire spine is interconnected in a complex manner, positional considerations and reporting should extend beyond neck position. Cord stretching will alter the strain distribution in the constituent spinal tissues, which could have a large implication on the mechanical properties of the gray and white matters, which are both hyper viscoelastic materials.

Stretching of the spinal cord also depends on the level of injury since flexion-induced stretching is non-uniformly distributed along the cervical spine (35, 37). It has been previously shown that neck flexion causes the highest strain at the C4-C6 segment (53). Flexion of the head and neck stretches the cord and dura by 7.5% over the C3-C6 segments, and 4.2% over the C6-T2 segments (35). The magnitude of cord stretching at each segment is correlated with the rotation of the corresponding vertebral body. It was previously reported that a 65-degree neck flexion angle was non-uniformly distributed in the cervical region, with vertebral rotation angles between 40° and 53° at C2, 32° and 47° at C3, 22.5° and 33° at C4, 10° and 23° at C5, 2.5° and 15° at C6, and (-3°) and 9° at C7 (50).

Since neck position changes the amount of stretching in the cord, and the corresponding strain density in the constituent tissues, it is expected that the stiffness of the white and gray matter would change with neck position. The position-induced stretching of the spinal cord has larger implications for white matter, which consists of unidirectionally-oriented myelinated fibers. Flexion of the neck straightens these undulated fibers (10, 21, 27, 29, 54, 55), which further increases the stiffness of the white matter (56–58). These changes in stiffness would alter the level of deformation experienced by the tissues under an applied load, changing the tissue damage patterns observed in the cord despite applying a consistent impact protocol.

**Figure 2:**
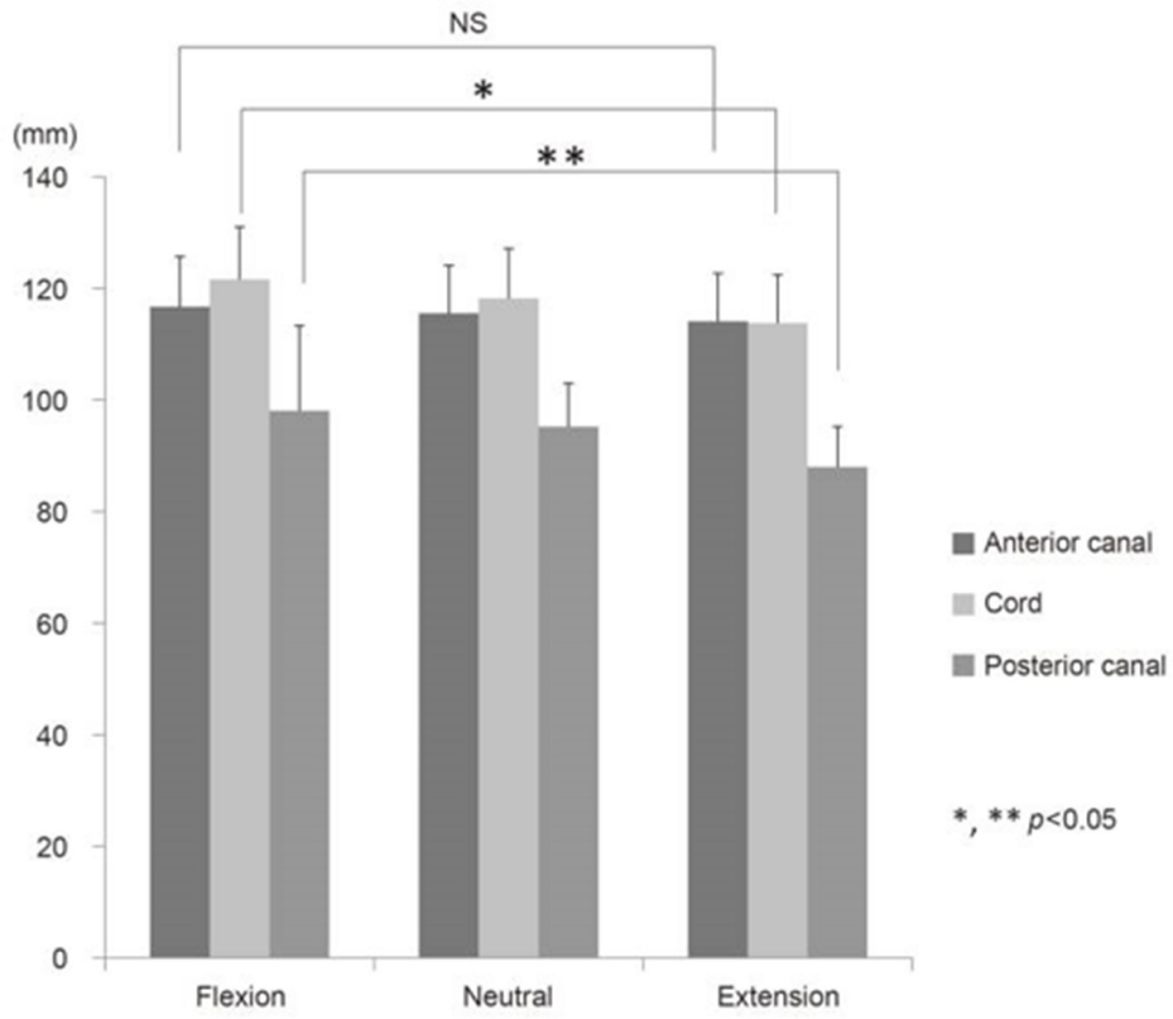
The effect on mechanical properties: changes in the length of the human spinal cord under various neck positions, reproduced from Endo et al. (32).

Cord stretching under flexion is also non-uniform in the dorsoventral axis (see Figure 2), and the dorsal surface of the cord experiences a higher strain than the ventral surface, creating further local variations in the strain within the constituent tissues (59). This disparity would cause the white and gray matter to exhibit anisotropic properties in the dorsoventral direction. In contusion models of animal spinal cord injury, the impact occurs on the dorsal side of the cord and the injury extends within the cord in the caudal and rostral directions, where the spinal cord experiences more stretching in flexion. Therefore, consistent positioning of the head and neck in cervical contusion injuries is important to ensure the repeatability of the impact as well as the transmission of loading through the tissue resulting in damage.

## 4. Future work

There is sufficient evidence to suggest that inter-subject positional differences could be a source of variability in cervical contusion models of animal spinal cord injury. Placing the head in flexion increases the mediolateral diameter of the cord, decreases the dorsoventral diameter of the cord, and places the cord closer to the ventral surface of the canal. The decreased amount of cerebrospinal fluid protecting the cord from the canal could lead to higher impact forces during the injury. The transverse widening of the cord could be beneficial in increasing the surface area available for contact with the impactor; however, placing the cord in flexion substantially increases the pretension in the cord, making it more susceptible to lateral slippage during a unilateral contusion injury. Comparatively, placing the cord in extension makes the cord rounder, providing more lateral space in the canal and potentially resulting in more lateral sliding. However, in extension, the cord experiences lower pretension, making it behave like a loose yarn making it less likely to slip laterally during an impact. The range of motion of each animal will affect positioning in either flexion or extension. For consistency in contusion impacts, based on the current knowledge, it is recommended that the head and neck be placed in a neutral position.

For comparison between studies, it would be beneficial to include quantitative details on the surgical position of animals in experimental studies. These should include details of neck and head position, such as whether the neck was placed in flexion, neutral, or extension position during the injury and care should be taken to position all animals in the same manner. Studies should also report how the limbs of the animals are positioned during the contusion injury and care should be taken to align the spine and pelvis of the animal to minimize rotation and lateral movements in the spine despite stabilizing it longitudinally.

Providing positioning details is vital in experimental studies on contusion models of cervical spinal cord injury. As more research groups begin conducting these experiments in large animals such as pigs and non-human primates, there is a need to control sources of variability so that experimental results are predictable. In addition, computational models can be used to quantify the effect of animal head and neck position on the biomechanics and outcomes in contusion SCI models. These investigative studies will provide quantitative guidance on the degree of variability introduced from variations in positioning, and the precision required for surgical positioning in these injury model systems, to establish the proper surgical methods underlying the evidence for contusion injury experiments from mimicked clinical settings.

## 5. Conclusions

The prevalence and severity of cervical spinal cord injuries demands focus on establishing effective treatment options. Animal models continue to play a critical role in the preclinical testing of promising therapies. Establishing consistent protocols for contusion experiments provides a better avenue for comparison between studies, and to ensure predictable and repeatable results.

Contusion experiments have been examined to minimize inter-subject variability attributed to factors including subject specifications, such as animal sex, weight, and age, and impact protocols, such as impactor velocity, force, impulse, or depth. However, animal positioning during contusion injuries has not been investigated as a source of variability and is often under-reported in studies. Neck position has previously been identified as an important factor influencing spinal cord injury biomechanics, mechanisms, and outcomes in humans; however, it has not been examined in animal models.

An extensive review of the literature of human studies showed that neck position, such as flexion or extension, has significant implications for the cervical spinal cord. This impact can be summarized into three main effects:

1. Neck position alters the rostral/caudal position of the spinal cord within the canal and could affect the resulting neurological level of injury.
2. Neck position alters the morphology of the spinal cord within the canal and could affect the distribution of tissue damage across the cord.
3. Neck position alters the mechanical properties of the constituent spinal tissues and could affect the resulting distribution of damage within the gray and white matters of the cord.

The large range of motion of the cervical spine makes it possible to achieve a broad range of positions using a skull clamp or stereotaxic frame during the surgery, which can propagate into variability in contusion biomechanics and resulting functional outcomes. This makes it vital to consider animal surgical positioning as a source of variability during a cervical contusion surgery, and report animal and subject-specific positioning in experimental studies.

## Author contributions

All authors contributed to manuscript revision, read, and approved the submitted version.
